# Ge nanopillar solar cells epitaxially grown by metalorganic chemical vapor deposition

**DOI:** 10.1038/srep42693

**Published:** 2017-02-17

**Authors:** Youngjo Kim, Nguyen Dinh Lam, Kangho Kim, Won-Kyu Park, Jaejin Lee

**Affiliations:** 1 Department of Electrical and Computer Engineering, Ajou University, Suwon 16499, Korea; 2 Korea Advanced Nano Fab Center, Suwon 16229, Korea; 3 Department of Physics, Hanoi National University of Education, Hanoi 100000, Vietnam

## Abstract

Radial junction solar cells with vertically aligned wire arrays have been widely studied to improve the power conversion efficiency. In this work, we report the first Ge nanopillar solar cell. Nanopillar arrays are selectively patterned on p-type Ge (100) substrates using nanosphere lithography and deep reactive ion etching processes. Nanoscale radial and planar junctions are realized by an n-type Ge emitter layer which is epitaxially grown by MOCVD using isobutylgermane. *In situ* epitaxial surface passivation is employed using an InGaP layer to avoid high surface recombination rates and Fermi level pinning. High quality n-ohmic contact is realized by protecting the top contact area during the nanopillar patterning. The short circuit current density and the power conversion efficiency of the Ge nanopillar solar cell are demonstrated to be improved up to 18 and 30%, respectively, compared to those of the Ge solar cell with a planar surface.

Vertically aligned microwire^1^,^2^,^3^ and nanowire^4^,^5^,^6^,^7^,^8^,^9^,^10^,^11^ (or nanopillar) arrays have been employed for photovoltaic applications to improve the power conversion efficiency (*PCE*). The wire-array solar cells promise an improved performance compared to planar solar cells due to reduced surface reflection and enhanced carrier collection efficiency^3^. The combined effects of intrinsic anti-reflection and efficient excitation of resonant modes at the nanopatterned surface induce superior absorption characteristics^12^. Furthermore, the photogenerated carriers can diffuse to the junction with minimal recombination because photons are absorbed mainly along the vertical direction and carriers are collected along the radial direction in the wires^3^,^8^,^13^,^14^. The diffusion length of the photo-generated carriers is less dependent on the depth of the optical absorption in the wire-array solar cells. Increased photoactive junction area due to the high surface to volume ratio of the wires also contribute to the enhanced carrier generation and collection efficiency^15^.

The wire-array solar cells have been fabricated using various materials such as Si^1^,^2^,^3^,^4^,^5^, GaAs^6^,^7^,^9^, InP^10^,^11^, and III-nitride^16^. However, there is no report on nanowire Ge solar cells which would be used for various photovoltaic and thermophotovoltaic applications^17^. Narrow bandgap (0.66 eV) Ge solar cells have been employed as a bottom subcell in the InGaP/InGaAs/Ge triple-junction solar cells which have achieved very high efficiencies over 40%^18^,^19^. Stand-alone Ge solar cells have also been used for thermophotovotaic systems as a receiver, which use the narrow band radiation spectrum originating from a heat source^20^. Nanostructured Ge solar cells with higher efficiencies could make better the availability of these kind of device applications.

In this work, we report on the first Ge nanopillar (NP) solar cell. The Ge NP arrays are selectively patterned on Ga doped p-type Ge (100) substrates using nanosphere lithography, which is cost-effective compared to conventional electron-beam lithography^11^,^21^,^22^. During the nanopatterning process, the top contact area is protected by photoresist to use the conventional ohmic metal instead of transparent conducting oxide (TCO) which can increase series resistance and absorption losses^23^,^24^. On the p-type Ge NP template, an n-type Ge emitter, an n-type InGaP window, and n-type GaAs ohmic layers are grown by a low pressure metalorganic chemical vapor deposition (MOCVD). Radial and planar junctions are realized during the epitaxial growth of the n-type Ge emitter layer. The InGaP window layer is exploited as an *in-situ* epitaxial surface passivation layer to alleviate the high surface recombination rates and Fermi level pinning^25^. The Ge NP solar cells are fabricated by photolithography, metal evaporation, rapid thermal annealing, wet chemical etching, and back-end processes^26^,^27^. Characteristics of the fabricated Ge NP solar cells are investigated under air-mass 1.5 global (AM1.5G) illuminations (see Methods).

## Results

### Selective patterning of Ge NP arrays for metal contact

In general, transparent conducting oxides (TCOs) such as indium tin oxide (ITO)^1^,^6^,^7^,^8^,^9^,^10^,^11^ or aluminum zinc oxide (AZO)^3^,^8^ have been used for the top contact materials of the wire-array solar cells. The simple encapsulating method using TCO, however, is known to cause a transmission loss of 10–20% and a reduction of light trapping effects at the nanowire arrays^7^. Schottky contact formation at the TCO/semiconductor interface is another problem that forces the wire-array solar cells to be exposed under high annealing temperatures of 550–700 °C which, in turn, result in the degradation of epitaxial wafer^9^,^28^,^29^. To exploit the conventional metal contact, we separated the metal contact area, grid and bus line, from Ge NP arrays.

**Figure 1:**
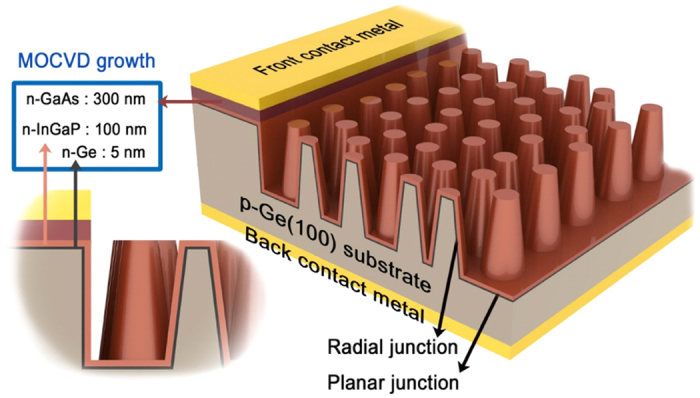
A schematic diagram of the Ge NP solar cell. (not to scale) The Ge NP solar cell consists of three parts including metal contact area, radial junction area, and planar junction area. An n-type Ge emitter layer (5 nm), an n-type InGaP window layer (100 nm), and an n-type GaAs ohmic layer (300 nm) are grown by MOCVD on the p-type Ge NP templates. The ohmic layer on the junction area is selectively removed during the device fabrication process.

A schematic diagram of the Ge NP solar cell is shown in Fig. 1. The Ge NP solar cell consists of three parts including metal contact area, radial junction area, and planar junction area. The Ge NP arrays are selectively patterned except the metal contact area. By employing the metal contact separation method, conventional ohmic metal structures can be used for the top contact without n-GaAs degradation^30^,^31^. In this work, AuGe(80 nm)/Ni(20 nm)/Au(400 nm) films are deposited by an e-beam evaporator as the top contact metal. The Ge NP solar cells are annealed at a temperature of 385 °C for 30 s to achieve an ohmic interface between the metal contact and the n-type GaAs layer.

**Figure 2:**
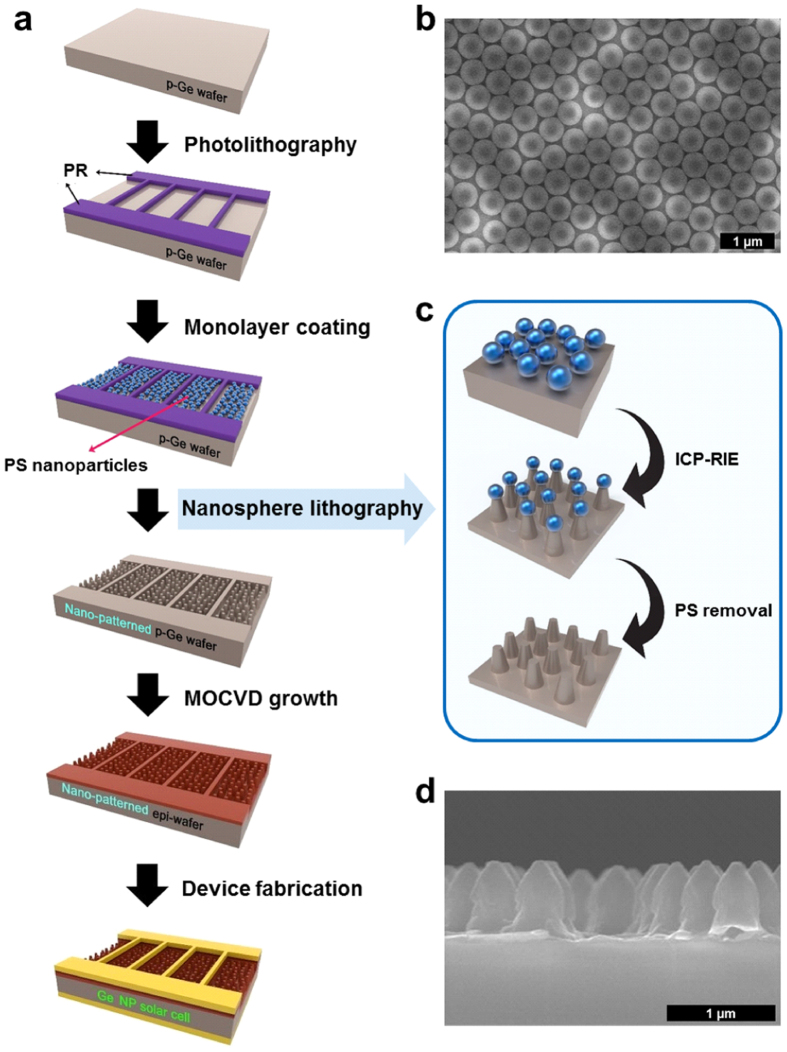
Illustrations of the selective patterning process. (not to scale) (**a**) Top contact arrays are patterned by photolithography on a p-type Ge wafer to protect the metal contact area with PR. A nearly close-packed nanosphere monolayer is coated on the aperture area, as shown in (**b**) the upper SEM image, using PS nanoparticles with a diameter of 500 nm. (**c**) The Ge NP arrays are patterned by nanosphere lithography using ICP-RIE and PS removal processes, as shown in (**d**) the lower SEM image. Finally, the Ge NP solar cell is fabricated by epitaxial growth and device fabrication processes.

The selective patterning process is illustrated in Fig. 2(a,c) and scanning electron microscope (SEM) images are shown in Fig. 2(b,d). First, top contact arrays are patterned by a photolithography process on a p-type Ge (100) substrate to protect the metal contact area with photoresist (PR). Each solar cell (5 × 5 mm^2^) has two bus lines and nineteen grid lines with length of 4 mm, width of 7 μm, and spacing of 250 μm. Second, polystyrene (PS) nanoparticles with a diameter of 500 nm are coated on the aperture area. A nearly close-packed PS nanosphere monolayer, as shown in Fig. 2(b), can be obtained by adjusting the hydrophilicity, the speed of spin coating, and the concentration of PS colloidal solution^22^. Third, the patterned Ge substrate is etched by an inductively coupled plasma reaction ion etching (ICP-RIE) system, as shown in Fig. 2(d). The heights of Ge NP arrays can be controlled by varying the etching time. After the dry etching process, residual PS nanoparticles and PR are removed by dipping into toluene and acetone in sequence. Finally, the Ge NP solar cell is fabricated by epitaxial growth using MOCVD and device fabrication processes.

**Figure 3:**
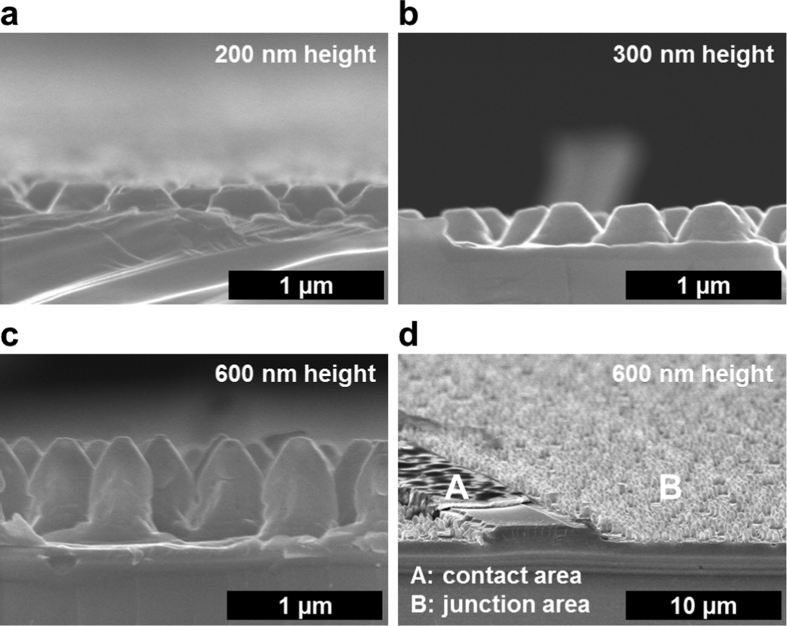
Cross-sectional SEM images of the Ge NP solar cells. The fabricated Ge solar cells have (**a**) 200, (**b**) 300, and (**c**) 600 nm height NP arrays. The Ge NP has conical shapes and the tip size becomes smaller when the etching depth is deeper. The metal contact area is successfully separated from the junction area as shown in a (**d**) 20 degree tilted image of the Ge solar cell with 600 nm height NP arrays.

The heights of Ge NP arrays are varied from 200 to 600 nm to find out the optimum nanostructure promising higher light absorption properties. Figure 3 shows the cross-sectional SEM images of the fabricated Ge solar cells with (**a**) 200, (**b**) 300, and (**c**, **d**) 600 nm height NP arrays. The Ge NP arrays have conical shapes and the tip size becomes smaller when the etching depth is deeper. The etching time is supposed to affect the cone-shape formation because the PS nanoparticles are also etched during the RIE process. The metal contact area can be successfully separated from the junction area as shown in Fig. 3(d), which is a 20 degree tilted SEM image of the Ge solar cell with 600 nm height NP arrays.

### Epitaxial growth of Ge NP solar cell structures using MOCVD

An n-type Ge emitter layer, an n-type InGaP window layer, and an n-type GaAs ohmic layer are grown by a low pressure MOCVD system on the p-type Ge NP templates as shown in Fig. 1 (see Methods). Radial and planar Ge p-n junctions are realized during the epitaxial growth of the n-type Ge emitter layer. The emitter thickness of 5 nm is selected to achieve a high *PCE* with a shallow junction depth (see Supplementary Information). Typically, the Ge p-n junctions are realized by phosphorus atomic diffusion into the p-type Ge substrates for stand-alone Ge solar cells^20^,^32^,^33^ and Ge based multi-junction solar cells^34^,^35^. Here, the Ge epitaxial growth technique using an isobutylgermane (IBuGe) in MOCVD is employed instead of the phosphorus diffusion process to obtain high quality radial and planar junction interfaces. IBuGe is a novel Ge metalorganic source which has high vapor pressure and is less toxic than germane (GeH_4_)^36^. Epitaxially grown Ge p-n junctions are expected to be well-defined with a high crystal quality^37^ and Ge solar cells with the improved junctions may achieve higher conversion efficiencies^38^,^39^.

The n-type InGaP window layer is grown on the Ge NP structure as a surface passivation layer to avoid from high surface recombination rates and Fermi level pinning^25^. The window thickness of 100 nm is selected to cover all the NP surfaces including the tips (see Supplementary Information). The surface passivation is an important process for the wire-array solar cells which have a large surface area-to-volume ratio^2^,^40^. The recombination of photogenerated carriers through the wire-array surface states can reduce the carrier diffusion length and, in turn, result in low efficiencies^41^. To achieve the surface passivation, amorphous silicon (a-Si) and hydrogenated amorphous silicon nitride (a-SiN:H) films are deposited for Si solar cells^2^,^3^, whereas an InGaP or AlGaAs epitaxial layer is grown for GaAs solar cells^9^,^25^. For Ge solar cells investigated in this study, the InGaP window layer is epitaxially grown to exploit the *in-situ* passivation which is one of the most effective way for the reduction of the surface state density^9^.

Finally, the n-type GaAs ohmic layer is grown for the ohmic contact formation between the top metal contact and the Ge NP solar cell structure. During the device fabrication process (see Methods), the GaAs ohmic layer on the junction area is selectively removed using a NH_4_OH:H_2_O_2_:H_2_O (2:1:10) etchant to avoid unintended optical losses.

**Figure 4:**
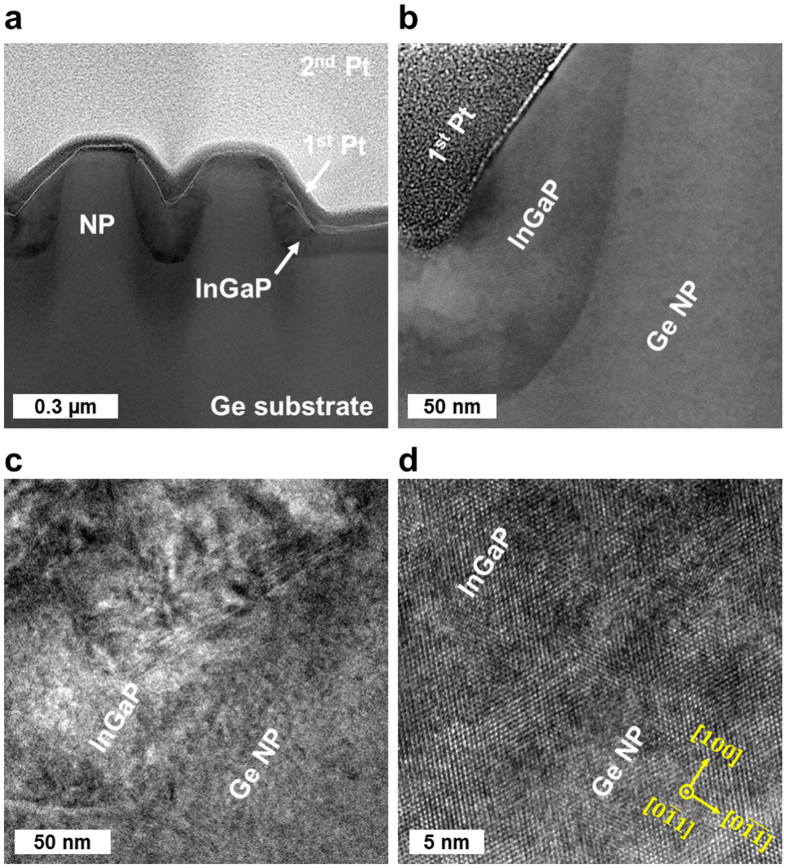
Cross-sectional TEM images of the Ge NP solar cell. The radial and planar junction structure of the Ge solar cell with 300 nm height NP arrays was investigated with a STEM system with a Cs corrector in <011> projection. The InGaP/Ge interface is well-defined as shown in the (**a**,**b**) STEM images. The different growth conditions around the NP arrays might induce the non-uniform InGaP thickness and several crystal defects, which are observed in some parts of the InGaP layer as shown in the (**c**) TEM and (**d**) high-resolution TEM images.

The fabricated Ge NP solar cell structure is investigated by a scanning transmission electron microscope (STEM) system with a Cs corrector in <011> projection. The TEM specimen is prepared by a focused ion beam (FIB) system and the first and second Pt protection layers are deposited on the surface using an electron beam and an ion beam evaporation, respectively. Figure 4 shows the (**a**, **b**) STEM, (**c**) TEM, and (**d**) high resolution TEM images of the Ge solar cell with 300 nm height NP arrays. The InGaP/Ge interface is well-defined as shown in the STEM images. The thickness of the InGaP window layer, however, is different depending on the position around the NP. The thickness of the InGaP layer located near the side of the Ge NP is 150 nm which is much thicker than the one on the planar junction area. Especially, 15 nm thick InGaP layers are observed on the tip of the Ge NP. This phenomenon is similar to the growth rate enhancement for selective area growth on patterned substrate using a dielectric mask^42^. The interfacial roughness of the epitaxial layers is determined by the surface kinetics such as absorption, migration, desorption, reaction, dissociation, and incorporation^43^. The different gas phase diffusion flux of the InGaP precursors due to the nano-patterned surface profile can change the surface kinetic processes and result in the non-uniform InGaP layer formation on the NP arrays^42^. The different growth conditions around the NP arrays might induce detrimental defects, such as vacancy, dislocation, and stacking fault, which are observed in some parts of the InGaP layer as shown in the TEM images.

### Photovoltaic characteristics of the Ge NP solar cells

**Figure 5:**
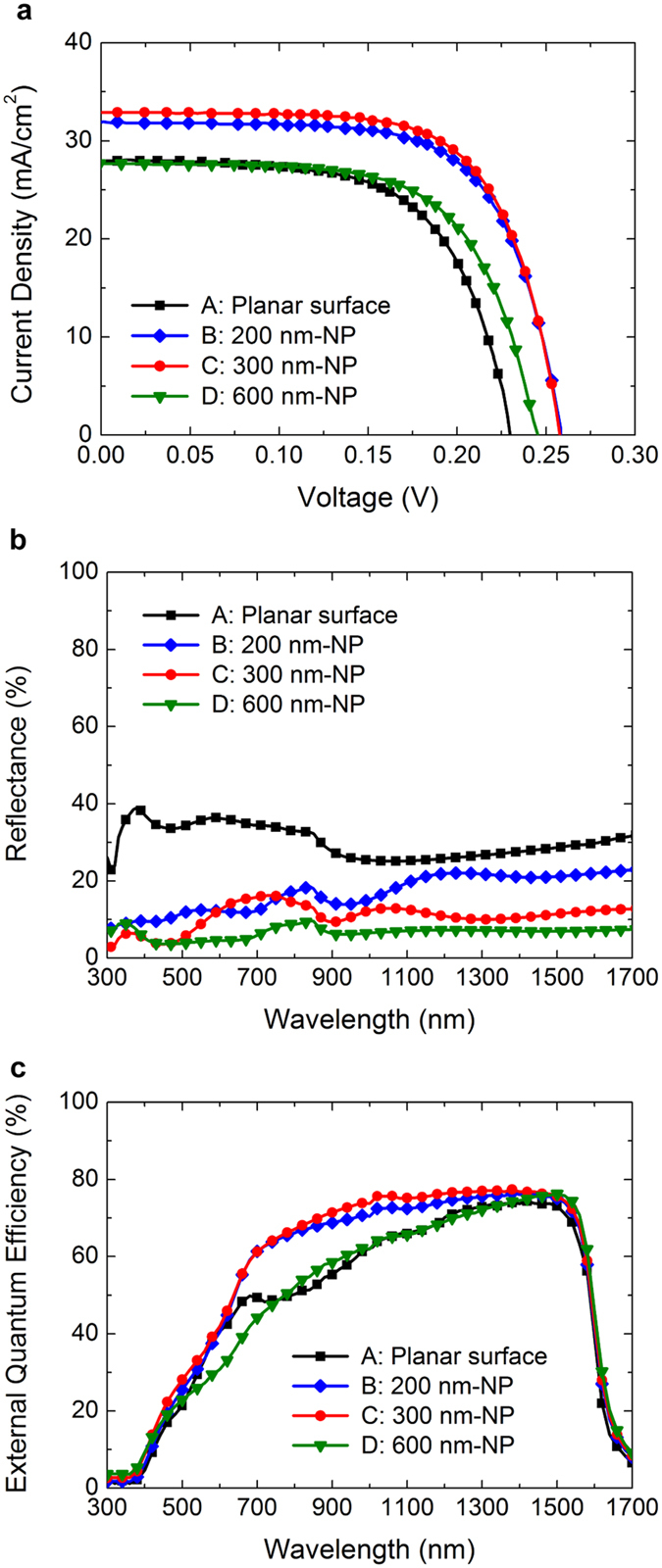
Device characteristics of the Ge NP solar cells. (**a**) The photovoltaic *J-V* curves under AM1.5 G illuminations, (**b**) surface reflectance spectra, and (**c**) EQE characteristics of the reference Ge solar cell A, which have a planar surface, and the Ge solar cell B, C, and D, which have 200, 300, and 600 nm height NP arrays, respectively.

The photovoltaic characteristics of the fabricated Ge NP solar cells are investigated with a class A solar simulator under AM1.5 G illuminations (see Methods). The current density-voltage (*J*-*V*) curves of the Ge solar cells with and without NP arrays are shown in Fig. 5(a) and the photovoltaic device parameters are summarized in Table 1. The short circuit current density (*J*_sc_) and *PCE* were corrected based on the external quantum efficiency (EQE) measurement. The reference Ge solar cell A, which has an all planar surface with a 100 nm thick InGaP layer and without anti-reflection coating (ARC) layers, is included here for comparison. The Ge solar cell B, C, and D, which have 200, 300, and 600 nm height NP arrays, respectively, show higher *PCE*s compared to the reference cell A. The increases of all the device parameters, such as the *J*_sc_, open circuit voltage (*V*_oc_), and fill factor (*FF*) all contribute to the *PCE* enhancement as shown in Table 1. The Ge solar cell B, which has 200 nm height NP arrays, shows the best *PCE* of 5.86% with *V*_oc_ of 0.26 V, *J*_sc_ of 31.89 mA/cm^2^, and *FF* of 0.7068. Especially, the *J*_*sc*_ of the solar cell B is 18% higher than that of the reference cell A which has a planar surface. The enhancement of the *J*_sc_ can be attributed to the reduced surface reflection and enhanced carrier collection efficiency. The *V*_oc_ and *FF* increase logarithmically with increasing photocurrent if dark current remains constant^44^. The higher *FF* also means that the Ge NP solar cell could have higher shunt resistance than the Ge solar cell with a planar surface as shown in Fig. 5(a).

When the height of Ge NP arrays becomes 600 nm, however, all the device parameters are degraded. The degradation can be attributed to the surface damages induced by the RIE process, which can result in plasma induced damages and surface contaminations because of ion bombardment, radiation induced bonding changes, and charge buildup^45^. It is the evidence that the *V*_oc_ is degraded as the height of Ge NP arrays increases from 200 to 600 nm as shown in Table 1. The longer RIE etching time for the Ge solar cell D may induce more damages at the p-n junction interface compared to the Ge solar cell B and C. In photovoltaic devices, the RIE induced surface damage causes significant degradation of the internal quantum efficiency (IQE) which reflects the charge separation and collection efficiency of the photogenerated carriers^46^. Further study including low-temperature *V*_oc_ analysis will be necessary to fully understand the effect of the RIE damage on the solar cell performances.

### Spectral responses of the Ge NP solar cells

The spectral responses of the fabricated Ge NP solar cells are investigated with a UV-VIS-NIR spectrophotometer and a solar cell quantum efficiency measurement system. Figure 5(b) shows the measured reflectance spectra of the Ge solar cells with and without NP arrays. The reduced reflection at the surface of the Ge NP solar cells is clearly observed in the measured wavelength range of 300 to 1700 nm. The light trapping effect is improved more when the height of NP arrays increase as shown in Fig. 5(b). The Ge solar cell D with 600 nm height NP arrays shows the lowest reflectance spectrum and the average reflectance is about 77% lower than that of the reference cell A with planar surface. At the surface of the Ge NP solar cell, the refractive index changes gradually due to the cone shaped NP arrays, whereas that across the air/InGaP planar interface changes of abrupt from 1.0 to 3.6 (at 650 nm)^47^. The dramatic decrease in the optical reflection of the Ge NP solar cell is mainly due to the refractive index which can be regarded as an infinite series of ARC layers^22^,^48^.

The measured EQE of the Ge solar cells with and without NP arrays are shown in Fig. 5(c). The Ge solar cell B and C, which have 200 and 300 nm height NP arrays, respectively, show higher EQEs compared to the reference cell A with planar surface in the wavelengths from 660 to 1580 nm. However, the EQE degradation of the Ge solar cell D with 600 nm height NP arrays is observed especially in the short wavelength range of 520 to 740 nm, which is affected by the quality of the window and emitter layers in the solar cells^44^. The RIE induced surface damage can result in the poor EQE response in the UV-visible region^46^. These observed results are in good agreement with the *J*_sc_ values in Table 1. Further detailed device physics of radial junction NP solar cells can be found in previous works focused on theoretical analysis and modeling^12^,^49^.

## Discussion

The *PCE* of the Ge single-junction solar cells can be improved by employing the radial and planar junction nanostructures based on vertically aligned NP arrays. When the height of NP arrays increase, the reflectance at the top surface of the Ge NP solar cells decreases gradually in the whole wavelength range of 300 to 1700 nm. The enhanced light absorption results in the higher EQE and *J*_sc_ compared to those of the Ge solar cell with planar surface. Especially, high quality Ge solar cell structures with NP arrays can be realized by the epitaxial Ge growth and the selective patterning processes.

Most of nanowire solar cells are suffering from lower efficiencies compared to state-of-the-art devices which have a planar surface^4^,^5^,^6^,^7^,^8^,^9^,^31^. In contrast, we report on the first successful Ge NP solar cell fabricated with a high efficiency of 5.86%. It is worth noting that the efficiency was achieved by the nanostructured Ge solar cell without ARC layers in this study. Moreover, it is expected that the device performance would be improved further when the thickness of the InGaP window layer is reduced. The carriers generated in the InGaP layer may not be converted into current and subjected to be recombined. Therefore, much higher EQEs can be achieved by reduction of the absorption loss in the wavelengths below 660 nm, if it is possible to successfully reduce the InGaP thickness from 100 to 30 nm.

Another limitation of the efficiency enhancement in the Ge NP solar cell is the degraded p-n junction interface due to the RIE induced damage. The Ge solar cell with 600 nm height NP arrays can absorb the incident light more efficiently due to the lowest reflection characteristics. However, the Ge NP solar cell, which is exposed to the RIE process for a long time, suffers from the surface recombination and the leakage current at the damaged p-n junction interface. The degradation of device performance may be minimized by the optimization of the RIE etching parameters. Various surface treatments such as RTA, thermal oxidation, and wet chemical etching can also reduce the detrimental effect of the RIE damaged layer^46^.

In conclusion, we report the first nanostructured epitaxial Ge solar cell with vertically aligned NP arrays. The highest *PCE* of 5.86% can be achieved from the Ge solar cell with 200 nm height NP arrays under AM 1.5 G illuminations. Reduced surface reflectance due to the graded refractive index of the Ge NP arrays results in improved light absorption. The *J*_sc_ of the Ge NP solar cell can increase up to 18% compared to that of the Ge solar cell with a planar surface. The metal contact separation method promises the high quality Ge NP solar cells without TCO.

## Methods

### Epitaxial growth

Single-junction Ge solar cell structures were epitaxially grown on the p-type Ge NP templates, as shown in Fig. 1, in a horizontal reactor of a low pressure MOCVD system (AIXTRON: AIX200/4 RF). Trimethylgallium (TMGa), trimethylindium (TMIn), arsine (AsH_3_), and phosphine (PH_3_) were used as group III and V precursors, whereas IBuGe is used as a Ge precursor. Silane (SiH_4_) was used as a dopant source for the n-type InGaP and GaAs layers which have doping levels of 1 × 10^18^ and 3 × 10^18^ cm^−3^, respectively. Palladium-diffused ultra-high-purity hydrogen (H_2_) was used as a carrier gas with a total flow of 15,000 sccm. The reactor pressure was fixed at 160 mbar and the growth temperature was kept at 670 °C. The growth rates of the Ge, InGaP, and GaAs layers were 6.8, 3.3, and 6.2 Å/s, respectively. Before the growth, the native oxide on the surface was removed by thermal annealing at 670 °C for 5 min with an AsH_3_ flow of 30 sccm.

### Device fabrication

After the epitaxial growth, the n-type ohmic metal films (AuGe/Ni/Au) were deposited on the metal contact area by using an electron-beam (e-beam) evaporator (Ulvac: EI-5). To obtain designed grid metal pattern, photolithography is performed on a mask aligner (EVG: EVG620) and a track system (SVS: MSX1000) which can perform PR coating, baking, and development. A metal lift-off process is performed on an organic wet-bench using acetone, isopropylalcohol (IPA), and deionized (DI) water. The p-type ohmic metal films (Ti/Pt/Au) were deposited on the bottom surface without any patterns by using the e-beam evaporator. For ohmic contact formation, the metal structure is annealed by using a rapid thermal annealing (RTA) system (NYM Tech: RTA150H-SVP1) under nitrogen (N_2_) ambient. Each solar cell device was isolated by employing a conventional mesa etching process and separated by using a dicing saw system (Disco: DAD3350). Finally, the solar cell was mounted on a metal printed circuit board (PCB) by using silver paste (CANS: P-100). The top contact was connected by thin gold wires using a wedge-wedge wire bonder (West Bond: 7476D).

### Photovoltaic device characterization

Photovoltaic *J-V* characteristics were measured by a class A solar simulator (Wacom: WXS-220S-L2) which has a xenon lamp and three halogen lamps for a high precision. First, the artificial spectral irradiance was calibrated for an hour by using a Si reference cell to make sure AM1.5 G 1 sun (1000 W/m^2^) illuminations. Second, the current density was corrected based on the *J*_*sc*_ calculated from the EQE measurement to eliminate the error caused by the uncertainty in the artificial spectrum. During the test, the stage temperature was kept at 25 °C by using a chiller. The test was performed in air without any masks because the device area was defined by the mesa etching and sawing processes. At least 10 solar cells were tested for each structure to make sure the device parameters and the standard deviation of the *PCE* was below 2%. EQE characteristics were measured by a solar cell quantum efficiency measurement system (PV measurements: QEX7). Si and Ge photodiodes were used for the system calibration in the wavelength range of 300−1000 nm and 1001−1700 nm, respectively. The estimated error in the EQE measurement amounts to ± 1−2%. Surface reflectance at the interface between the air and the top surface of the solar cell was investigated with a UV-VIS-NIR spectrophotometer (Varian: Cary 5000).

## Additional Information

**How to cite this article**: Kim, Y. *et al*. Ge nanopillar solar cells epitaxially grown by metalorganic chemical vapor deposition. *Sci. Rep.* **7**, 42693; doi: 10.1038/srep42693 (2017).

**Publisher's note:** Springer Nature remains neutral with regard to jurisdictional claims in published maps and institutional affiliations.

## Supplementary Material

Supplementary Information

## Figures and Tables

**Table 1:** Photovoltaic device parameters of the Ge NP solar cells under AM1.5 G illuminations.

Ge NP height (nm)	*PCE* (%)	*V*_oc_ (V)	*J*_sc_ (mA/cm^2^)	*FF*
A: N/A	4.51	0.24	27.96	0.6716
B: 200	5.86	0.26	31.89	0.7068
C: 300	5.82	0.25	32.89	0.7079
D: 600	4.55	0.24	27.68	0.6842
