# Supplementary material for: Ge nanopillar solar cells epitaxially grown by metalorganic chemical vapor deposition
Source: Sci Rep. 2017 Feb 17;7:42693. doi: 10.1038/srep42693 (PMC5314339; doi:10.1038/srep42693)
Supplement: Supplementary Information [file srep42693-s1.pdf]

Supplementary Information:

Ge nanopillar solar cells epitaxially grown by metalorganic chemical vapor deposition

Youngjo Kim[1,2], Nguyen Dinh Lam[1,3], Kangho Kim[1,2], Won-Kyu Park[2], and Jaejin Lee[1,*]

[1]Department of Electrical and Computer Engineering, Ajou University, Suwon 16499, Korea
[2]Korea Advanced Nano Fab Center, Suwon 16229, Korea
[3]Department of Physics, Hanoi National University of Education, Hanoi 100000, Vietnam
[*]Corresponding author: jaejin@ajou.ac.kr

Single-junction Ge solar cell structures consisting of n-type Ge emitter, n-type InGaP window, and n-type GaAs cap layers have been grown by MOCVD on p-type Ge (100) substrates. Before the epitaxial growth, nanopillar (NP) arrays (200 nm in diameter and 300 nm in height) were patterned on the Ge substrates to realize the Ge NP solar cell structures.

Ge NP solar cells with different emitter layers in thickness were investigated to improve the carrier collection efficiency. The thickness of the n-type Ge emitter layer was varied from 0 to 500 nm, whereas those of the InGaP window layer and the GaAs cap layer were kept at 100 and 300 nm, respectively. It was found that the thinner emitter layer is helpful to obtain higher device performance in the Ge NP solar cells. When the emitter thickness decreases from 500 to 5 nm, the power conversion efficiency (*PCE*) increased from 4.06 to 5.82 % as shown in Figure S1 (**a**) and Table S1. Especially, the short circuit current density (J_{sc}) was improved up to 27.9 %. The enhancement of J_{sc} could be attributed to the thinner emitter region which affects to the carrier collection efficiency. It is evidence that the Ge NP solar cell with a 5 nm thick n-type Ge emitter layer shows relatively higher external quantum efficiencies (EQE) in

the wavelengths below 1100 nm as shown in Figure S1 (**b**). Comparable results of conventional Ge planar solar cells are shown in Figure S2 and Table S2. Epitaxial n-type Ge emitter layers were grown on p-type Ge planar substrates with different thicknesses. 30 nm thick InGaP window and 300 nm thick GaAs cap layers were also grown on the Ge p-n junction structures. It could be found that a thinner emitter layer also contributes to achieve a higher efficiency for the planar cell. Relatively high *PCE* and J_{sc} are due to thinner window layer and MgF_2/ZnS anti-reflection coating (ARC) layers deposited on the top surface.

Ge NP solar cells with different window layers in thickness were investigated to reduce the optical loss in the window layer. We tried to reduce the thickness of the InGaP window layer to 30 nm, which is widely used for general III-V solar cells. However, the *PCE* decreased from 5.82 to 3.33 % as shown in Figure S3 (**a**) and Table S3. The EQE of the Ge NP solar cell with a 30 nm thick window layer was degraded in a broad wavelength regime from 600 to 1600 nm as shown in Figure S3 (**b**). These results could be attributed to the higher surface recombination velocity at the NP tips. It was found that the window thickness of 30 nm is not thick enough to cover all the NP surfaces of the Ge NP solar cells due to the different gas phase diffusion flux on the nanopatterned surfaces during the MOCVD growth. Comparable results of Ge solar cells, grown on planar substrates, are shown in Figure S4 and Table S4. The planar cell consists of an epitaxial n-type Ge emitter layer (5 nm), an InGaP window layer (30, 100 nm), a GaAs cap layer (300 nm), and MgF_2/ZnS ARC layers. In contrast, the Ge planar solar cell with a 30 nm thick InGaP window layer shows a relatively high efficiency due to improved reflection and absorption properties.

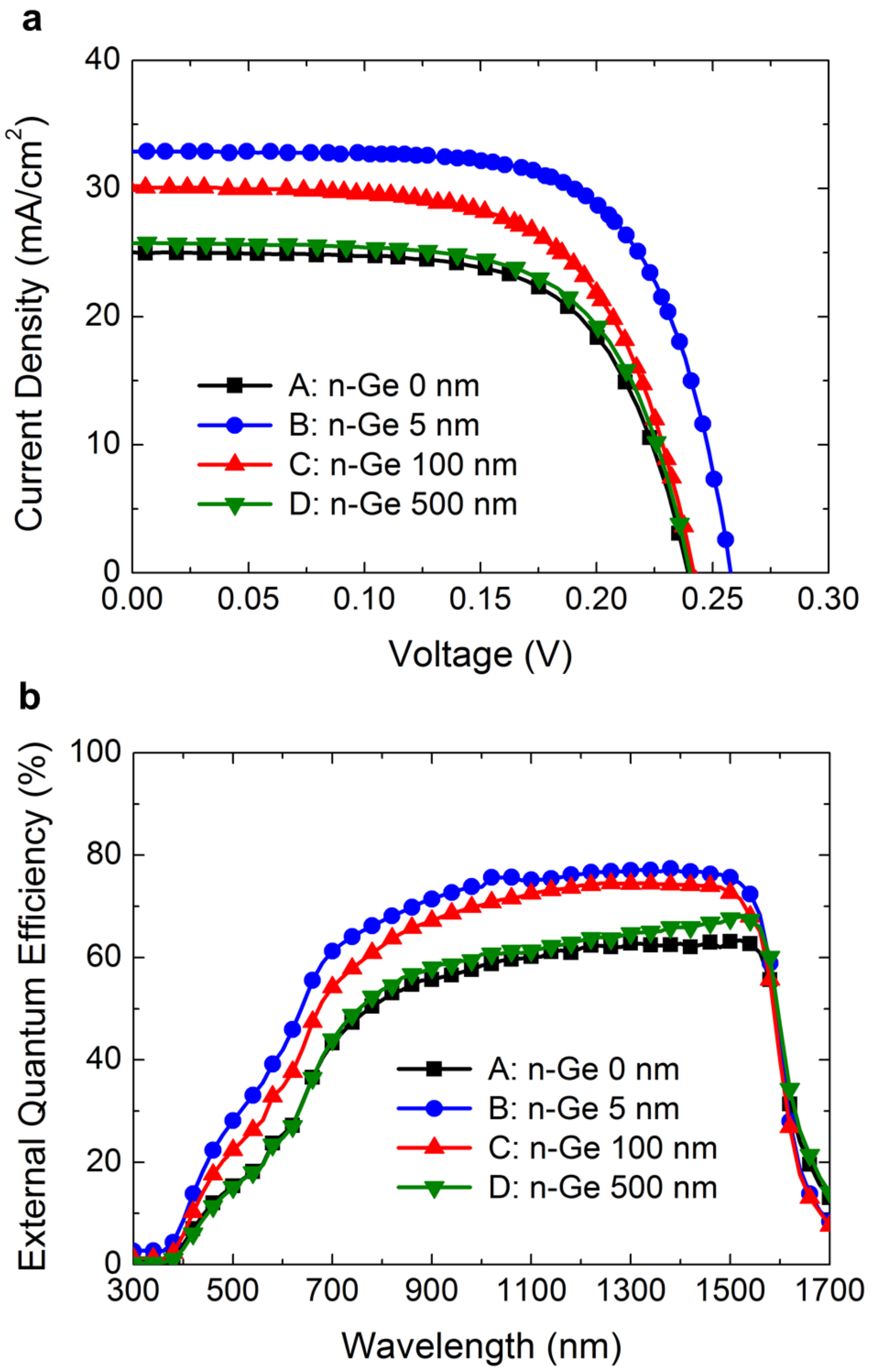

Supplementary Figure S1. (**a**) Photovoltaic *J*-*V* curves and (**b**) external quantum efficiencies of the Ge NP solar cells with different n-type Ge emitter layers in thickness. The InGaP window thickness was kept at 100 nm and the NP arrays were designed to be 200 nm in diameter and 300 nm in height.

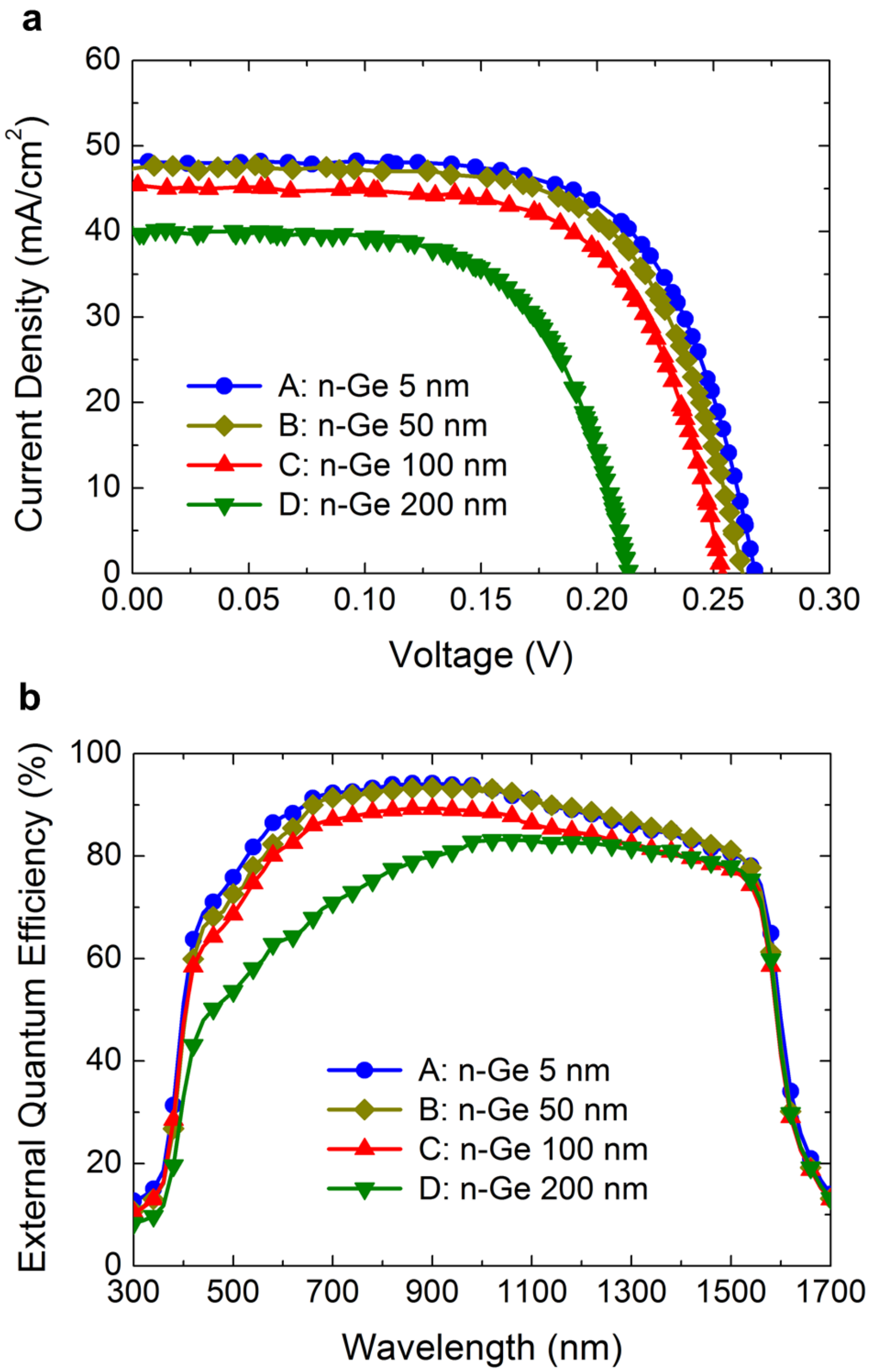

Supplementary Figure S2. (**a**) Photovoltaic *J-V* curves and (**b**) external quantum efficiencies of the Ge planar solar cells with different n-type Ge emitter layers in thickness. The InGaP window thickness was kept at 30 nm and MgF_2/ZnS ARC layers were deposited on the top surface.

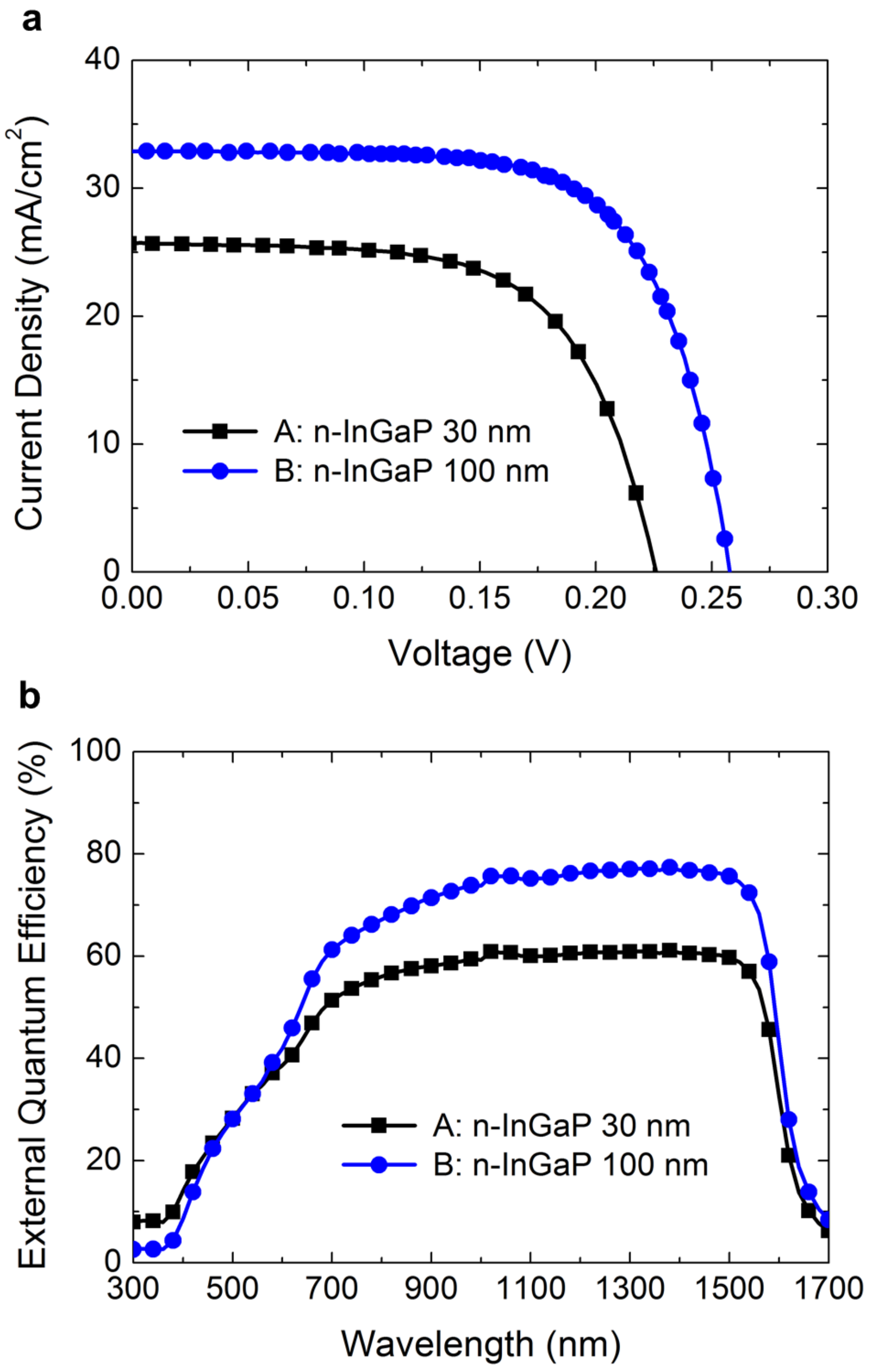

Supplementary Figure S3. (**a**) Photovoltaic *J*-*V* curves and (**b**) external quantum efficiencies of the Ge NP solar cells with different InGaP window layers in thickness. The n-type Ge emitter thickness was kept at 5 nm and the NP arrays were designed to be 200 nm in diameter and 300 nm in height.

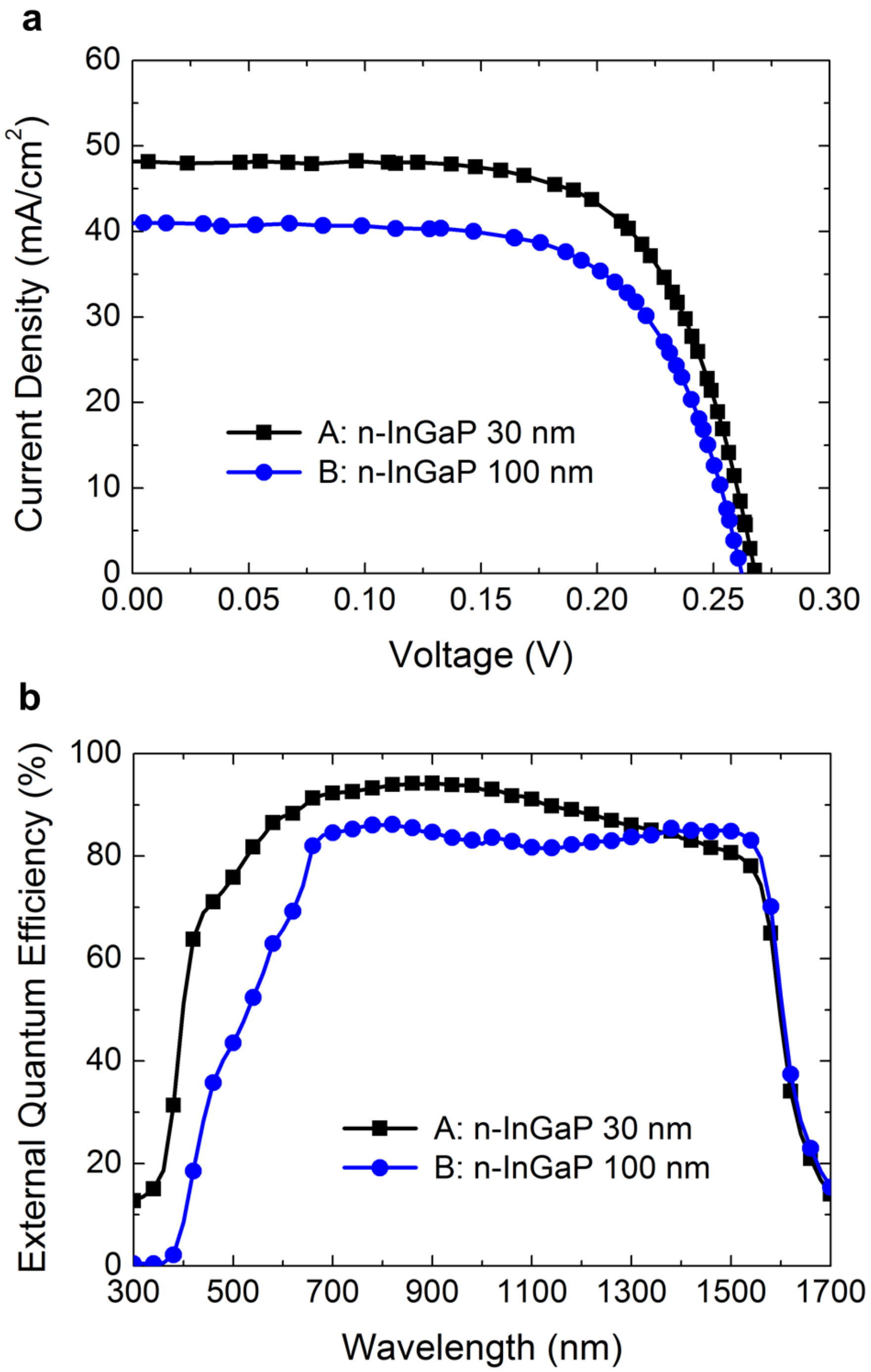

Supplementary Figure S4. (**a**) Photovoltaic *J*-*V* curves and (**b**) external quantum efficiencies of the Ge planar solar cells with different InGaP window layers in thickness. The n-type Ge emitter thickness was kept at 5 nm and MgF_2/ZnS ARC layers were deposited on the top surface.

Supplementary Table S1: Specific device parameters of the Ge NP solar cells with different n-type Ge emitter layers in thickness under AM1.5G illuminations. The InGaP window thickness was kept at 100 nm and the NP arrays were designed to be 200 nm in diameter and 300 nm in height.

	n-Ge thickness (nm)	*PCE* (%)	V_{oc} (V)	J_{sc} (mA/cm^2)	*FF*
A:	0	3.93	0.24	24.98	0.6560
B:	5	5.82	0.25	32.89	0.7079
C:	100	4.87	0.24	30.21	0.6723
D:	500	4.06	0.24	25.71	0.6582

Supplementary Table S2: Specific device parameters of the Ge planar solar cells with different n-type Ge emitter layers in thickness under AM1.5G illuminations. The InGaP window thickness was kept at 30 nm and MgF_2/ZnS ARC layers were deposited on the top surface.

	n-Ge thickness (nm)	*PCE* (%)	V_{oc} (V)	J_{sc} (mA/cm^2)	*FF*
A:	5	8.77	0.27	48.21	0.6735
B:	50	8.26	0.26	47.36	0.6709
C:	100	7.47	0.25	45.31	0.6595
D:	200	5.32	0.21	39.80	0.6368

Supplementary Table S3: Specific device parameters of the Ge NP solar cells with different InGaP window layers in thickness under AM1.5G illuminations. The n-type Ge emitter thickness was kept at 5 nm and the NP arrays were designed to be 200 nm in diameter and 300 nm in height.

	n-InGaP thickness (nm)	*PCE* (%)	V_{oc} (V)	J_{sc} (mA/cm^2)	*FF*
A:	30	3.33	0.23	27.50	0.5264
B:	100	5.82	0.25	32.89	0.7079

Supplementary Table S4: Specific device parameters of the Ge planar solar cells with different InGaP window layers in thickness under AM1.5G illuminations. The n-type Ge emitter thickness was kept at 5 nm and MgF_2/ZnS ARC layers were deposited on the top surface.

	n-InGaP thickness (nm)	*PCE* (%)	V_{oc} (V)	J_{sc} (mA/cm^2)	*FF*
A:	30	8.77	0.27	48.21	0.6735
B:	100	7.06	0.26	40.97	0.6628
